# XPS and structural studies of Fe_3_O_4_-PTMS-NAS@Cu as a novel magnetic natural asphalt base network and recoverable nanocatalyst for the synthesis of biaryl compounds

**DOI:** 10.1038/s41598-021-04111-z

**Published:** 2021-12-30

**Authors:** Homa Kohzadi, Mohammad Soleiman-Beigi

**Affiliations:** Department of Chemistry, Faculty of Basic Sciences, Ilam University, 69315-516 Ilam, Iran

**Keywords:** Chemistry, Nanoscience and technology

## Abstract

In this research, natural asphalt as a mineral carbonuous material was converted to sodium natural asphalt sulfonate (Na-NAS) and, then, was linked to Fe_3_O_4_ MNPs in order to synthesize the magnetic nanocatalyst. Afterwards, Cupper (I) and Cu (II) was grafted on Fe_3_O_4_-PTMS-NAS. Moreover, it is worth mentioning that the synthesized the novel magnetic nanocatalyst (Fe_3_O_4_-PTMS-NAS@Cu) was successfully used in Suzuki and Stille coupling reactions. The Fe_3_O_4_-PTMS-NAS@Cu MNPs were characterized by Fourier transform infrared spectroscopy (FT-IR), scanning electron microscopy (SEM), transmission electron microscopy (TEM), energy-dispersive X-ray spectroscopy (EDX), X-ray diffraction (XRD), thermogravimetric analysis (TGA), vibrating sample magnetometry (VSM), inductively coupled plasma (ICP), BET and X-ray photoelectron spectroscopy (XPS) analysis. Besides, sulfonation of natural asphalt, magnetization of catalyst, grafting of Cu (I) and Cu (II) to NAS and catalyst formation were investigated and proved carefully. This nanocatalyst can be comfortably separated from the reaction medium through an external magnetic field and can also be recovered and reused, while maintaining its catalytic activity.

## Introduction

In resent decade, organic carbonious supports have attracted much attention in chemistry because of some specific benefits like high surface area, stability in both acidic and basic mediums, low toxicity and cost, availability and flexibility with regards to pore size^1–7^. Introducing a new class and developing carbon-based supports are interesting for chemists as new research fields in catalysis, synthesis, methodology and engineering.

In this regard, natural asphalt has been sulfonated and applied as a heterogeneous carbon-based catalyst in some organic reactions. In our previously reported works, we successfully grafted copper and potassium on natural asphalt and studied their catalytic properties^8,9^. Natural asphalt is one of the carbon materials which often exists in underground mines, consisting of 70–80%wt carbon and 15%wt hydrogen^9^. In continuation of our recent research, in order to develop and extend NAS application as a new catalyst support, Cu-NAS was linked to Fe_3_O_4_ as magnetic nanoparticles.

Today, utilizing magnetic nanoparticles in the organic chemistry has been greatly increased because these nanoparticles can be easily separated from the reaction mixture, using an external magnet^10–13^. Moreover, magnetic nanoparticles have low toxicity^14,15^ and can be recovered and reused up to several runs.

**Scheme 1:**
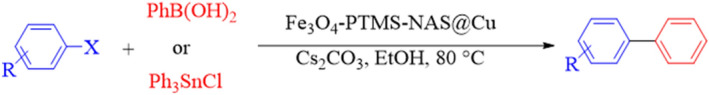
Suzuki and Stille coupling reactions using Fe_3_O_4_-PTMS-NAS@Cu MNPs.

The Suzuki and Stille^16–18^ are known as strong coupling reactions for carbon(SP^2^)-carbon bond formation and have been studied in the presence of various catalysts and transition metals. In this research, due to the advantages of natural asphalt, such as high activity, high surface area, low toxicity and specific features of natural asphalt, Fe_3_O_4_-PTMS-NAS@Cu as a new magnetic recyclable nanocatalyst is synthesized and applied in Suzuki and Stille coupling reactions (Scheme 1).

## Results and discussion

### Characterization of Fe_3_O_4_-PTMS-NAS@Cu MNPs

**Scheme 2:**
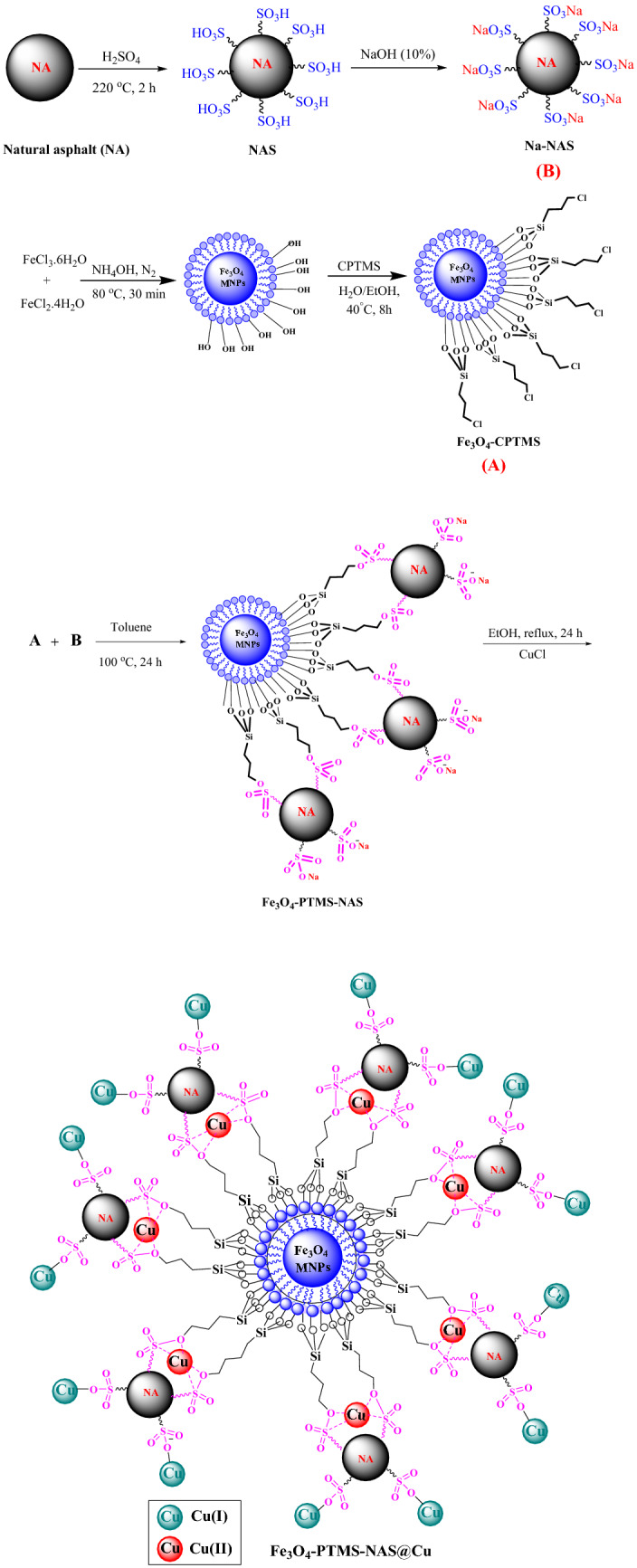
The general route for the synthesis of Fe_3_O_4_-PTMS-NAS@Cu MNPs.

The magnetic nanoparticles (Fe_3_O_4_-PTMS-NAS@Cu) were prepared using four-step process (Scheme 2). Natural asphalt sulfonic acid (NASA) was synthesized using the reaction of natural asphalt (NA) with concentrated sulfuric acid. Afterwards, sodium natural asphalt sulfonate (Na-NAS) was synthesized utilizing the NASA and NaOH solution^9,19^. Moreover, Fe_3_O_4_-CPTM was synthesized according to the literature^15^. Subsequently, in order to synthesize Fe_3_O_4_-PTMS-NAS, sodium natural asphalt sulfonate (Na-NAS) was immobilized on the surface of Fe_3_O_4_-CPTMS MNPs. In the final step, Fe_3_O_4_-PTMS-NAS@Cu MNPs were synthesized using Fe_3_O_4_-CPTMS-NAS and CuCl. In order to characterize Fe_3_O_4_-PTMS-NAS@Cu MNPs, FT-IR, SEM, EDX, XRD, TGA, VSM, ICP, BET and XPS techniques were used.

### Characterization of Fe_3_O_4_-PTMS-NAS@Cu MNPs

#### FT-IR analysis

**Figure 1:**
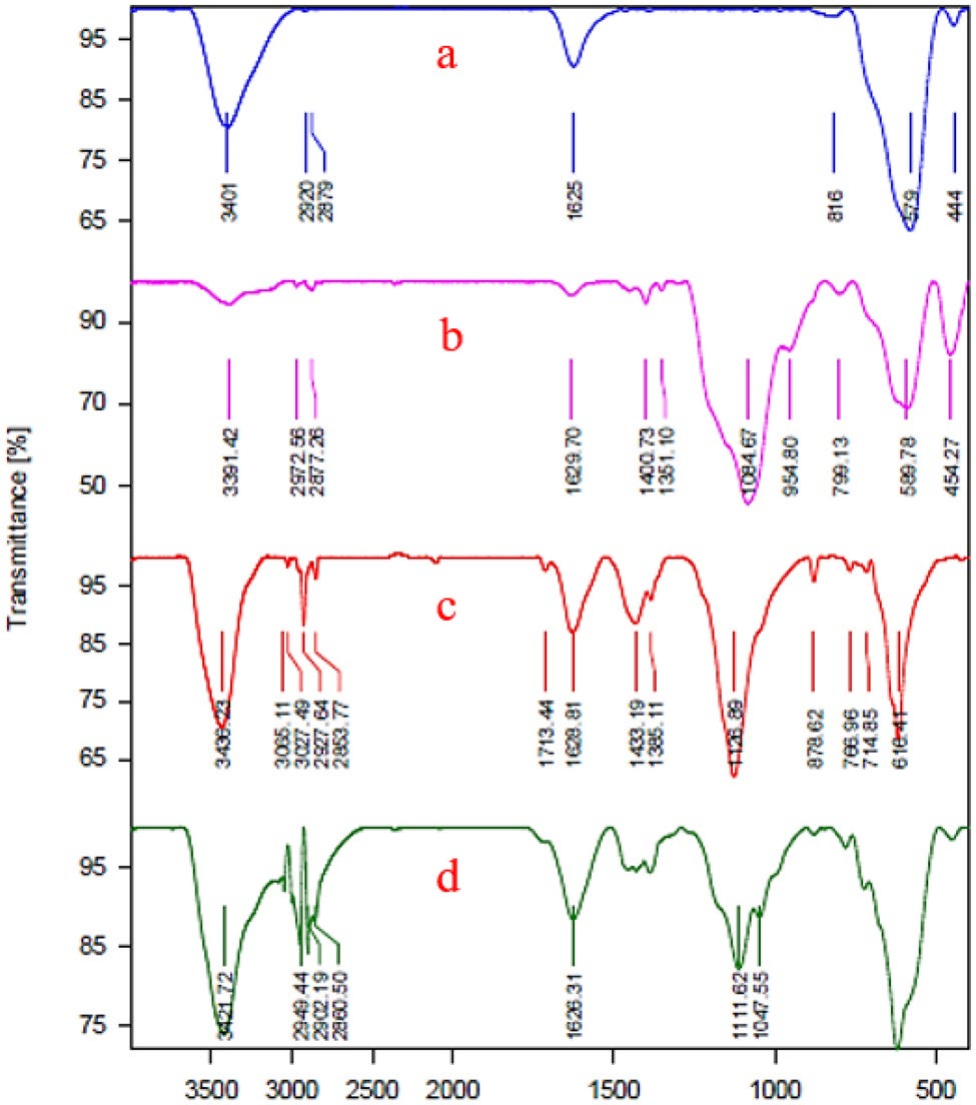
FT-IR spectra of Fe_3_O_4_ (**a**), Fe_3_O_4_-CPTMS (**b**), Fe_3_O_4_-PTMS-NAS (**c**) and Fe_3_O_4_-PTMS-NAS@Cu MNPs (**d**).

The FT-IR spectra of Fe_3_O_4_ (a), Fe_3_O_4_-CPTMS (b), Fe_3_O_4_-PTMS-NAS (c) and Fe_3_O_4_-PTMS-NAS@Cu (d) MNPs are shown in Fig. 1. The stretching vibrations at 579 cm^−1^ and 3401 cm^−1^ are associated with Fe–O and O–H bands in the structure of Fe_3_O_4_ (Fig. 1a). The peaks at 1084 cm^−1^ and 2877–2972 cm^−1^ are correlated to the stretching vibration of Si–O band and methylene groups (CH_2_) which confirmed the presence of 3-chloropropyltrimethoxysilane on the surface of Fe_3_O_4_ MNPs (Fig. 1b). The symmetric and asymmetric stretching vibrations at 610 cm^−1^ and 1128 cm^−1^ are related to the S=O in the SO_2_ bands, which confirm the successful immobilization and connection of the natural asphalt sulfonate on the surface of Fe_3_O_4_-CPTMS (Fig. 1c). The existence of Cu in the structure of the catalyst was approved through stretching vibration of S=O bands that appeared at 1111 cm^−1^, as this band shifts to lower frequency due to the grafting of cu on Fe_3_O_4_-PTMS-NASMNPs (Fig. 1d).

#### SEM and TEM analysis

**Figure 2:**
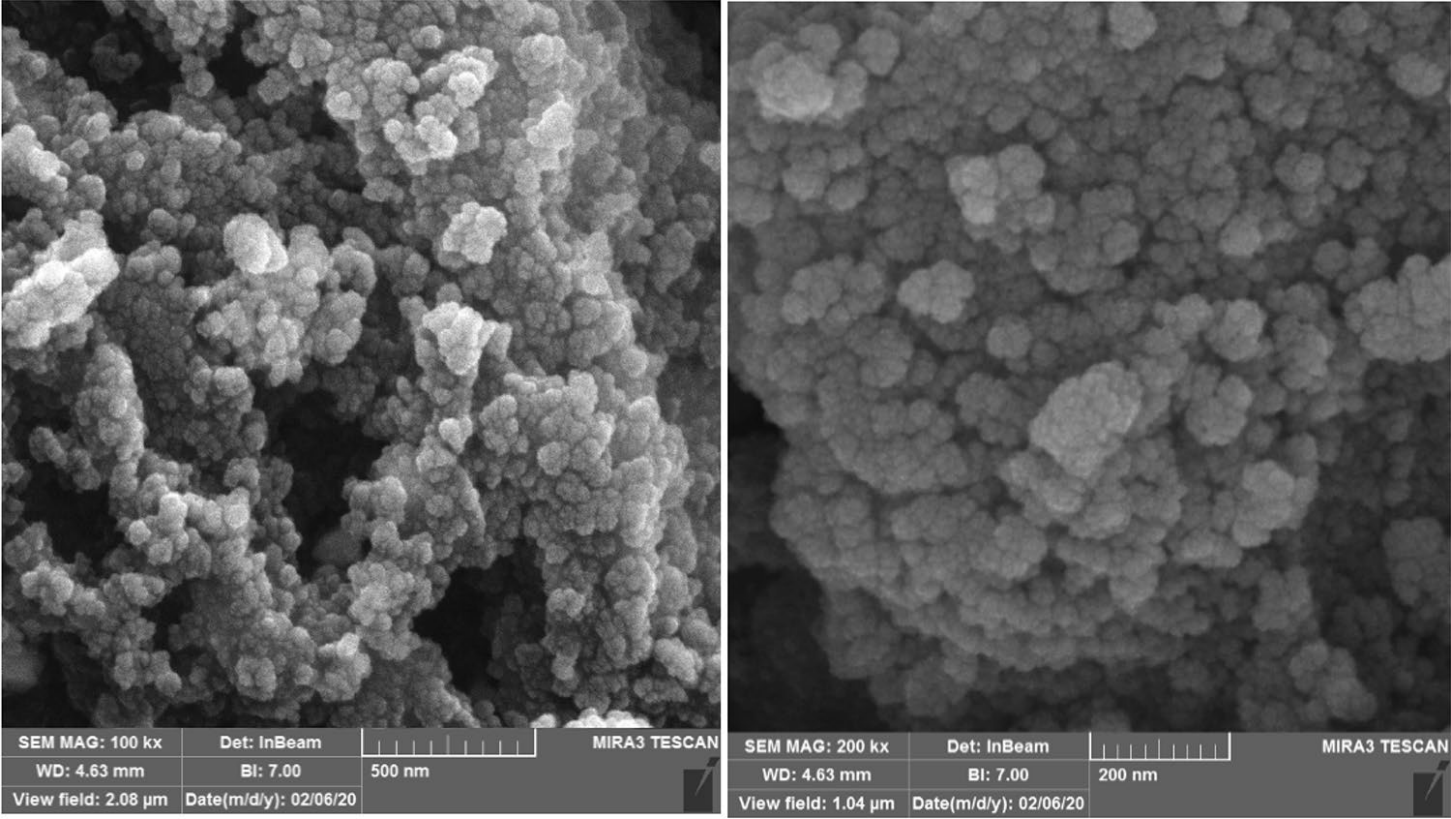
SEM images of Fe_3_O_4_-PTMS-NAS@Cu MNPs.

**Figure 3:**
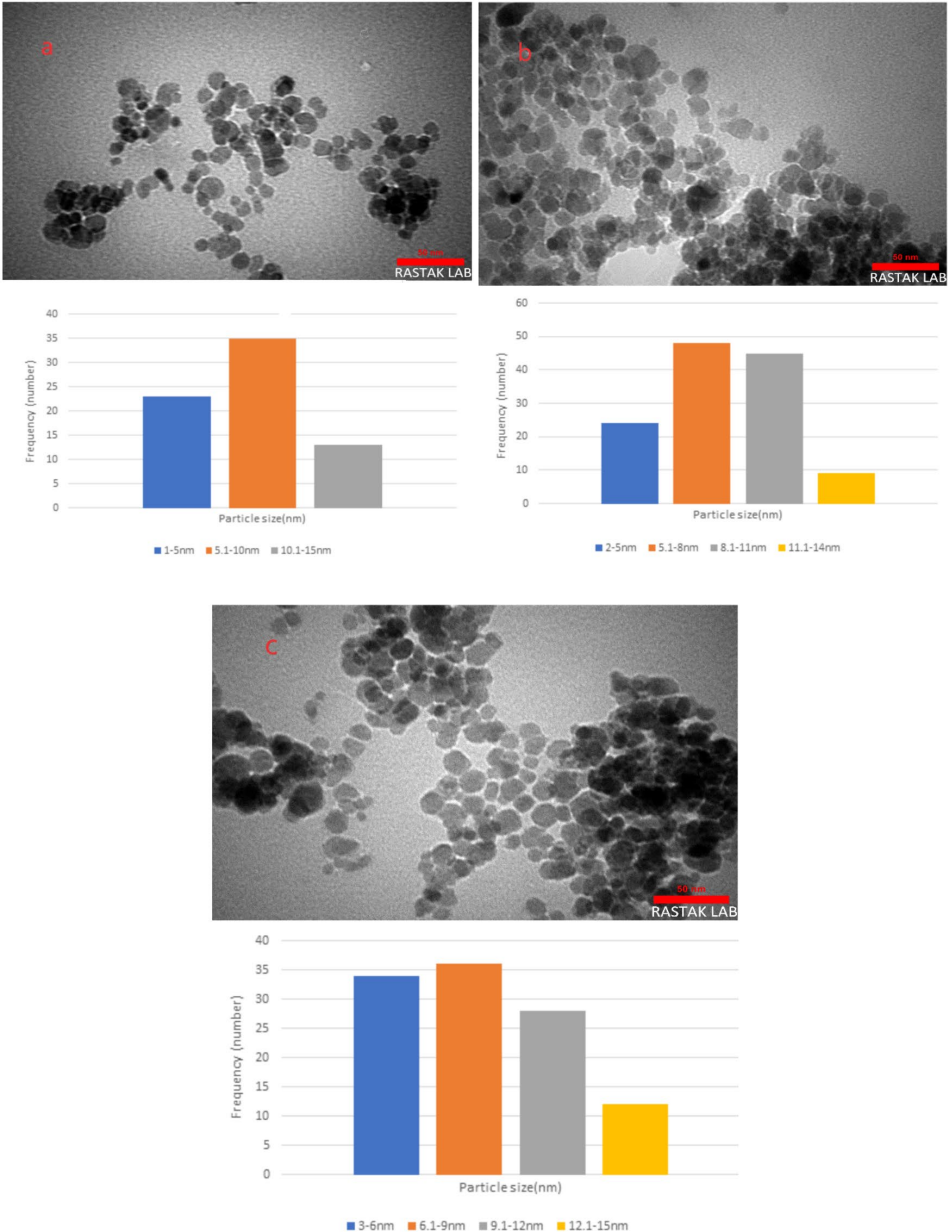
TEM images of Fe_3_O_4_ (**a**), Fe_3_O_4_-CPTMS (**b**) and Fe_3_O_4_-PTMS-NAS@Cu MNPs (**c**).

The morphology and size of Fe_3_O_4_-PTMS-NAS@Cu MNPs are determined by SEM and TEM analysis, as shown in Figs. 2 and 3. Based these images, nanoparticles are spherical in the shape and according to TEM images for the Fe_3_O_4_, Fe_3_O_4_-CPTMS and Fe_3_O_4_-PTMS-NAS@Cu MNPs, the average size of the nanoparticles are 5–10 nm, 5–8 nm and 6–9 nm respectively.

#### EDX analysis

**Figure 4:**
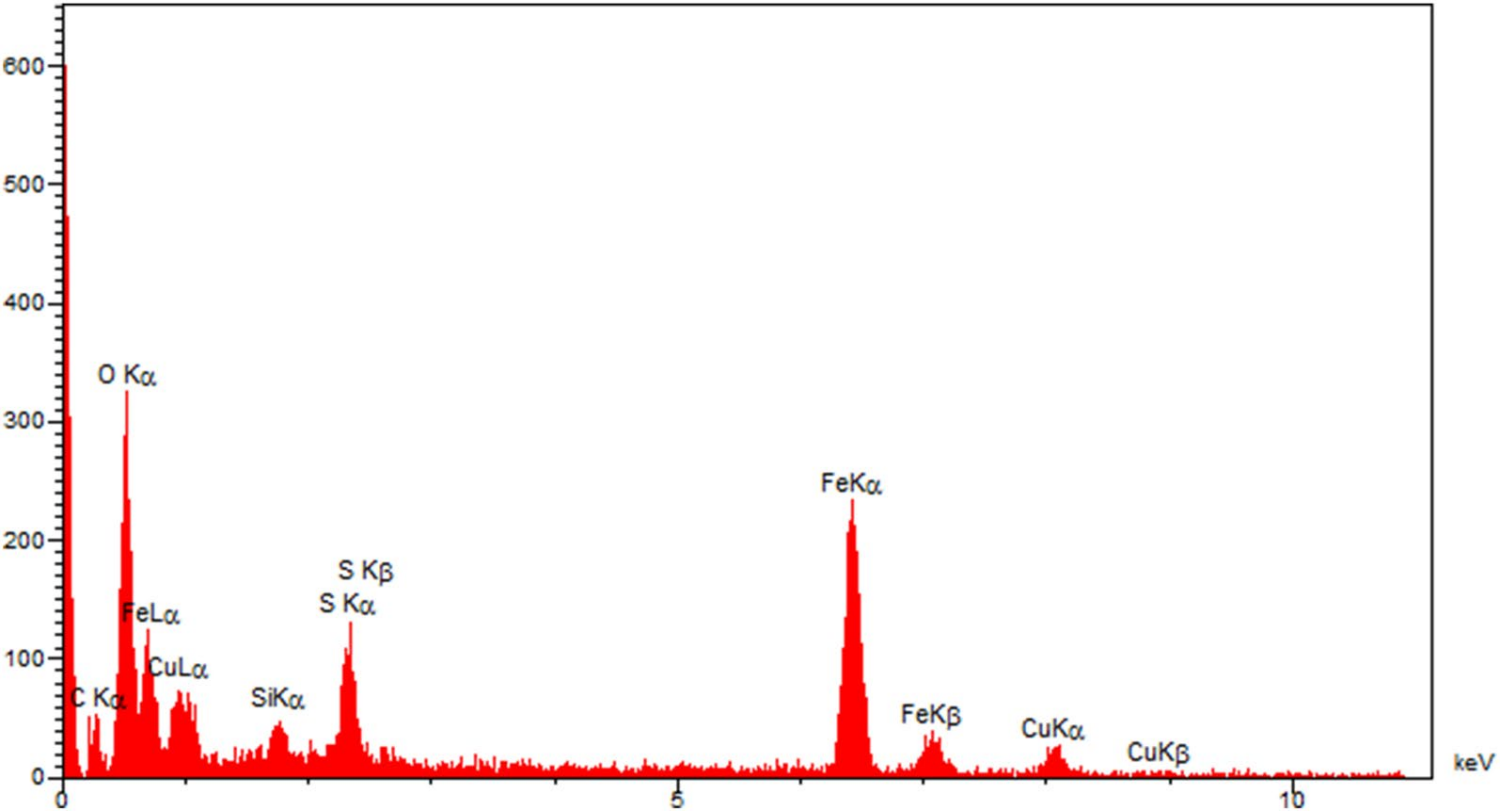
EDX spectrum of Fe_3_O_4_-PTMS-NAS@Cu MNPs.

**Figure 5:**
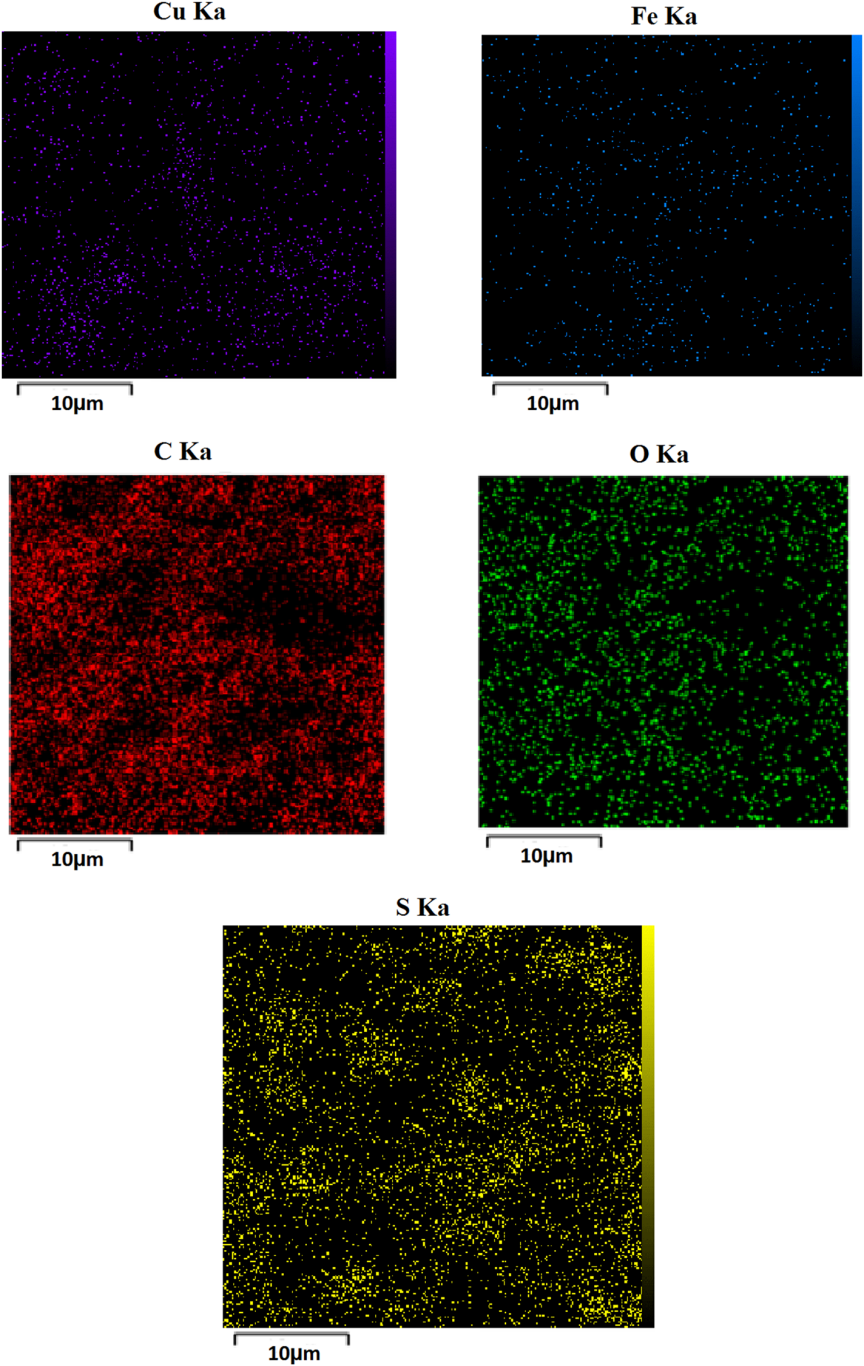
Elemental mapping of Fe_3_O_4_-PTMS-NAS@Cu MNPs.

One of the suitable analysis to determine elements, present in the structure of nanoparticles, is the energy-dispersive X-ray spectroscopy (EDS). The EDX spectrum of the Fe_3_O_4_-PTMS-NAS@Cu MNPs is demonstrated in Fig. 4, which confirms the presence of Fe, O, S and Cu elements in the structure of the catalyst and proved that the magnetic nanoparticle has been successfully synthesized. Moreover, the EDX mapping analysis (Fig. 5) obviously indicated the homogeneous distribution of all the elements.

#### XRD analysis

**Figure 6:**
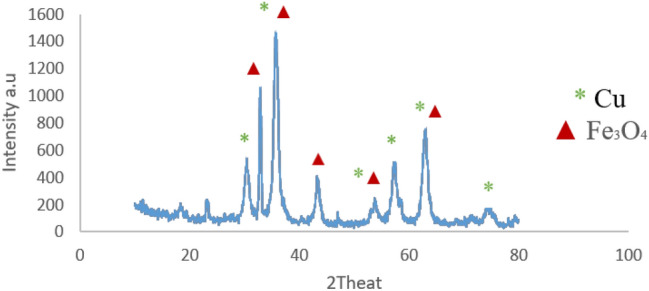
XRD pattern of Fe_3_O_4_-PTMS-NAS@Cu and the reference pattern according to ICSD:250540 and ICSD:75-0449.

The structure of Fe_3_O_4_-PTMS-NAS@Cu MNPs was investigated by x-ray diffraction (XRD). The XRD pattern of Fe_3_O_4_-PTMS-NAS@Cu MNPs is presented in Fig. 6, indicating that six peaks at 2Ɵ = 32.85°, 35.50°, 43.03°, 53.75° and 63.20° were related to Fe_3_O_4_ MNPs (ICSD:250,540). Moreover, 2Ɵ = 30.40°, 35.50°, 53.75°, 57.45°, 63.20° and 74.45° were related to Cu. These peaks proved that the structure of Fe_3_O_4_-PTMS-NAS@Cu MNPs is in good agreement with the standard pattern with the reference cards with numbers ICSD:250540 and ICSD:75-0449^20,21^. Also the crystal size was calculated according to Debye–Scherrer formula and the mean crystal size of Fe_3_O_4_ and Fe_3_O_4_-PTMS-NAS@Cu MNPs, was obtained 9.23 nm and 3.69 nm respectively.

#### TGA analysis

**Figure 7:**
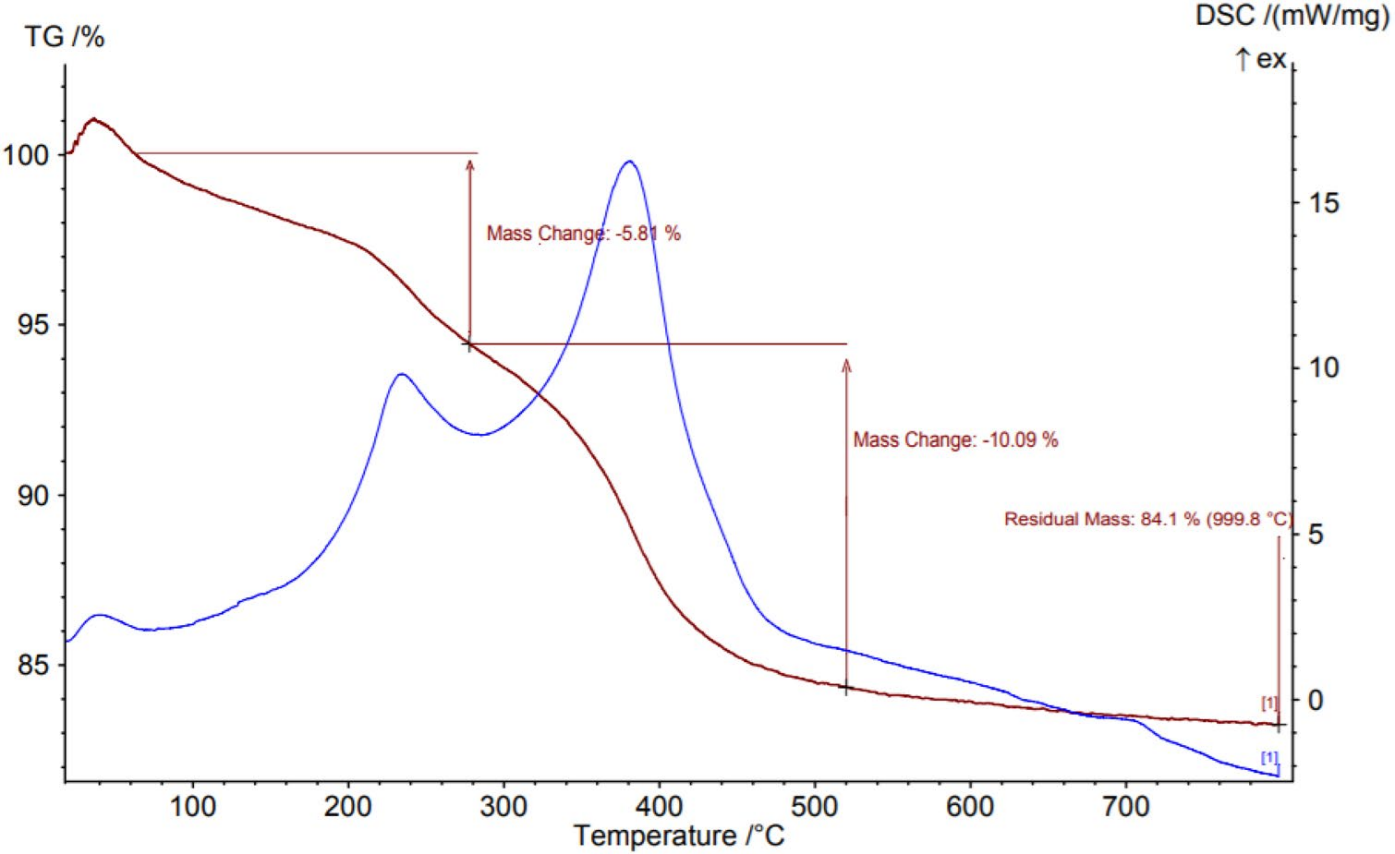
TGA curve of Fe_3_O_4_-PTMS-NAS@Cu MNPs.

Thermogravimetric analysis (TGA) and Differential Scanning Calorimetry (DSC) curves of the Fe_3_O_4_-PTMS-NAS@Cu MNP**s** in Fig. 7 indicates the two step weight loss, that the first weight loss (5.81%) was observed at below 260 °C which can be related to the removal of the physically adsorbed solvents and OH groups on the surface of the catalyst. Besides, the second weight loss (10%) occurred between 260 and 520 °C which is related to the decomposition of some organic groups on the surface of the catalyst such as polycyclic rings, R-SO_3_, etc.

#### VSM analysis

**Figure 8:**
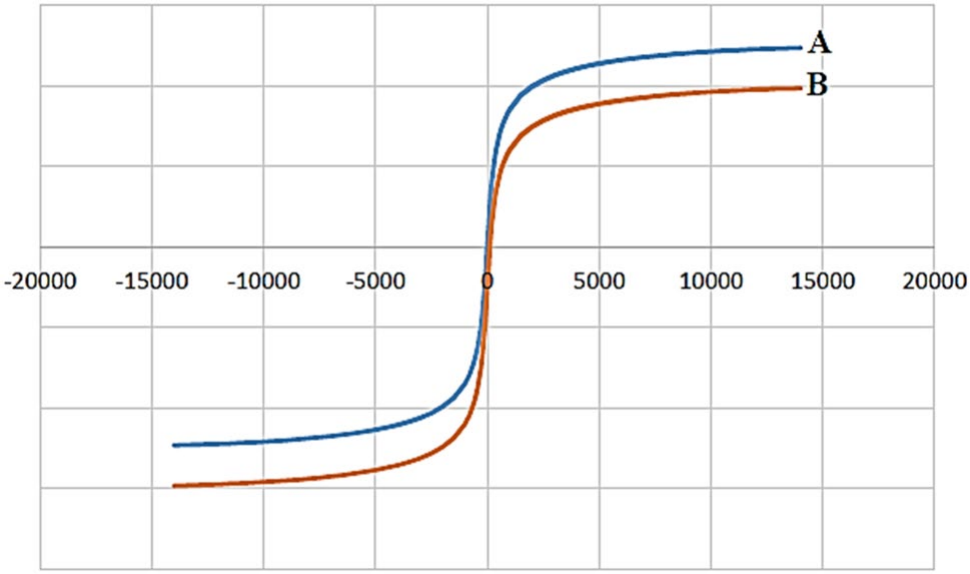
VSM curves of Fe_3_O_4_ (A) and Fe_3_O_4_-PTMS-NAS@Cu (B) MNPs.

The magnetic property of Fe_3_O_4_-PTMS-NAS@Cu MNPs was checked using vibrating sample magnetometry (VSM) technique at room temperature. Magnetization curves of Fe_3_O_4_ (A) and Fe_3_O_4_-PTMS-NAS@Cu MNPs (B) MNPs are illustrated in Fig. 8, illustrating that the amount of saturation magnetizations for Fe_3_O_4_ and Fe_3_O_4_-PTMS-NAS@Cu are 49 and 39 emu/g, respectively. Moreover, the decrease of saturation magnetization proved that the groups were immobilized on the surface of Fe_3_O_4_ MNPs and the catalyst was synthesized, successfully.

#### ICP analysis

In order to determine the amount of Cu on the surface of catalyst, the ICP atomic emission spectroscopy technique was used which indicated that the exact the amount of Cu, stabilized on surface of Fe_3_O_4_-PTMS-NAS MNPs, is found to be 1.70 mmol g^−1^.

#### BET analysis

The BET analysis shows the surface area of 37.39 and 18.18 for the Fe_3_O_4_-PTMS-NAS (A) and Fe_3_O_4_-PTMS-NAS@Cu (B), respectively. In addition, the average pore diameter and pore volume of Fe_3_O_4_-PTMS-NAS are 1.88 nm and 0.15 cm^3^ g^−1^ and for Fe_3_O_4_-PTMS-NAS@Cu are 1.66 nm and 0.064 cm^3^ g^−1^, respectively. The results are summarized in Table 1. Decrease of surface area, average pore diameter and pore volume for Fe_3_O_4_-PTMS-NAS@Cu MNPs, as compared to Fe_3_O_4_-PTMS-NAS MNPs, confirmed the successful grafting of copper on to Fe_3_O_4_-PTMS-NAS MNPs. Also according to the IUPAC classification, both of them show type-IV isotherm (defined by IUPAC), which are characterized as mesoporous materials.

**Table 1:** Textural properties of Fe_3_O_4_-PTMS-NAS and Fe_3_O_4_-PTMS-NAS@Cu.

Sample	SBET (m^2^ g^−1^)	Pore diam by BJH method (nm)	Pore vol (cm^3^ g^−1^)
Fe_3_O_4_-PTMS-NAS	37.39	1.88	0.15
Fe_3_O_4_-PTMS-NAS@Cu	18.18	1.66	0.064

#### XPS analysis

**Figure 9:**
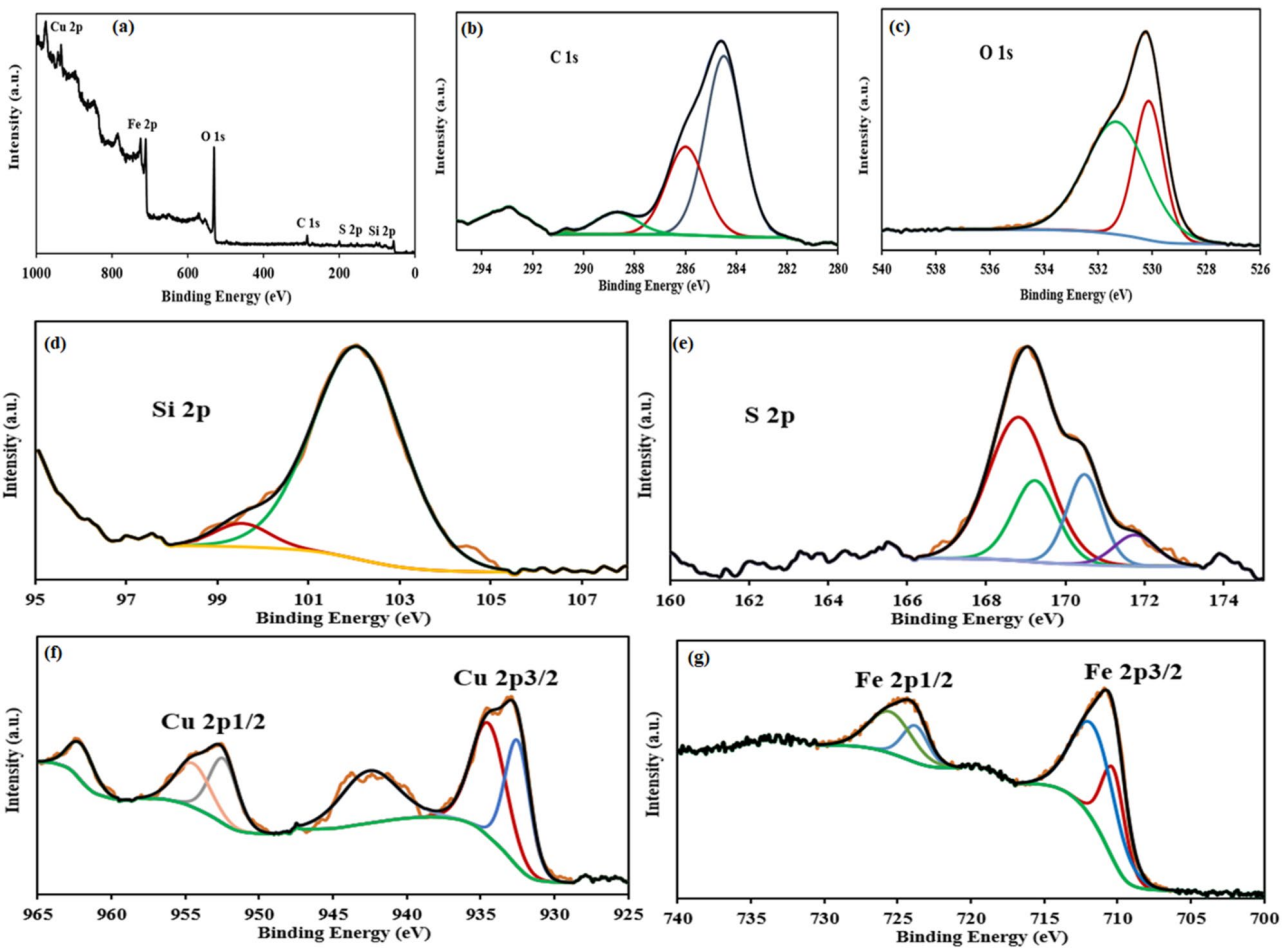
XPS curves of Fe_3_O_4_-PTMS-NAS@Cu MNPs.

In order to evaluate the oxidation states and elemental composition, the X-ray photoelectron (XPS) measurements were used. Figure 9 shows the XPS survey spectrum of Fe_3_O_4_-PTMS-NAS@Cu which clearly demonstrated the existence of C, O, Si, S, Cu, and Fe elements, as we excepted for the final product. The deconvoluted XPS spectrum of C 1 s (Fig. 9b) shows three fitting peaks at binding energies of 284.2, 286.3, and 288.9 eV, which can be related to C–C, C–O and C–S chemical bonds^22^. Three oxygen contributions (529.80, 530.9, and 531.31) eV can be assigned to O=S, C–O–C, and –COO– groups, respectively^22^. The deconvoluted XPS spectrum of S 2p would also reveal the presence of sulfur on the substrate surface. The S 2p_3/2_ peaks are located at 168.82 eV and 170.48 eV, corresponding to the sulfonic group (SO_3_H)^22^. These results confirmed that the natural asphalt has been sulfonation, successfully. The deconvoluted XPS spectrum of Cu 2p (Fig. 9f) shows two main peaks between 930 and 937 eV, related to Cu 2p_3/2_, and 950.0 and 962.0 eV related to the Cu 2p_1/2_. Eeach of these peaks consists of two main sub-peaks, corresponding to the monovalent Cu (I) and divalent Cu (II). As a results, the XPS spectrum data for Cu 2p confirmed that both Cu oxidation states (I and II) exist in the sample^22^. The de-convoluted Fe 1s XPS spectrum shows the fitted peaks at 711 eV (Fe 2p_3/2_) and 725 eV (Fe 2p_1/2_), confirming the successful functionalization of Fe_3_O_4_.

### Catalytic studies

After synthesis and analysis of the Fe_3_O_4_-PTMS-NAS@Cu MNPs structure, the activity of this nanocatalyst in the Suzuki and Stille coupling reactions was investigated. In this sense, the reaction of iodobenzene with phenylboronic acid (phenyl source) was chosen as model reaction. Besides, the effect of various parameters; including, solvent, temperature, base and various amounts of catalyst in the model reaction was studied. First, the effect of catalyst amount on the model reaction was studied. When the model reaction was examined in the absence of Fe_3_O_4_-PTMS-NAS@Cu, the reaction did not occur and the best results were obtained with 10 mg of Fe_3_O_4_-PTMS-NAS@Cu in dimethylformamide. Moreover, these processes were performed using series of solvents such as DMSO, PhCH_3_, H_2_O, DMF and EtOH and various bases. Accordingly, EtOH as solvent and Cs_2_CO_3_ as base showed the best results. In addition, different temperatures were studied on the model reaction, according to which 80 °C (reflux) showed the best results. The best yields were obtained in EtOH using 10 mg of Fe_3_O_4_-PTMS-NAS@Cu MNPs and (1 mmol) of Cs_2_CO_3_ at 80 °C for 90 min.

After obtaining the optimum conditions, some derivatives of Suzuki and Stille coupling using different aryl halides were synthesized whose results are demonstrated in Tables 2 and 3.

**Table 2:** Synthesis of biphenyl derivatives by Suzuki reaction in presence of Fe_3_O_4_-PTMS-NAS@Cu MNPs.


Entry	Aryl halide	Product	Yield (%)	Time (min)		M.P [ref.]
1	C_6_H_5_I	**2a**	94	90		63–65^23^
2	4-IC_6_H_4_OMe	**2b**	87	250		83–85^9^
3	4-IPhCH_3_	**2c**	92	190		45–46^23^
4	2-IPhCH_3_	**2d**	90	320		Oil^9^
5	BrC_6_H_5_	**2e**	88	100		63–65^23^
6	4-BrC_6_H_4_OMe	**2f**	82	310		83–85^9^
7	4-BrC_6_H_4_CN	**2g**	83	115		80–82^23^
8	4-BrPhCH_3_	**2h**	90	230		45–46^23^
9	4-BrC_6_H_4_NO_2_	**2i**	78	120		111–114^23^
10	2-Br-naphthalene	**2j**	65	700		100–102^9^

Reaction conditions: Aryl halide (1 mmol), phenylboronic acid (1 mmol), CS_2_CO_3_ (1 mmol), Fe_3_O_4_-PTMS-NAS@Cu MNPs (10 mg), EtOH (2 ml), reflux.

**Table 3:** Synthesis of biphenyl derivatives by the Stille reaction in presence of Fe_3_O_4_-PTMS-NAS@Cu MNPs.


Entry	Aryl halide	Product	Yield (%)	Time (min)	M.P [ref.]
1	C_6_H_5_I	**3a**	94	90	63–65^23^
2	4-IC_6_H_4_OMe	**3b**	90	210	83–85^9^
3	4-IPhCH_3_	**3c**	91	190	45–46^23^
4	2-IPhCH_3_	**3d**	88	310	Oil^9^
5	BrC_6_H_5_	**3e**	86	110	63–65^23^
6	4-BrC_6_H_4_OMe	**3f.**	86	300	83–85^9^
7	4-BrC_6_H_4_CN	**3 g**	85	100	80–82^23^
8	2-BrPhCH_3_	**3 h**	82	370	Oil^9^
9	4-BrPhCH_3_	**3i**	89	220	45–46^23^
10	4-BrC_6_H_4_NO_2_	**3j**	83	130	111–114^23^

Reaction conditions: Aryl halide (1 mmol), triphenyltin chloride (0.4 mmol), CS_2_CO_3_ (1 mmol), Fe_3_O_4_-PTMS-NAS@Cu MNPs (10 mg), EtOH (2 ml), reflux.

**Scheme 3:**
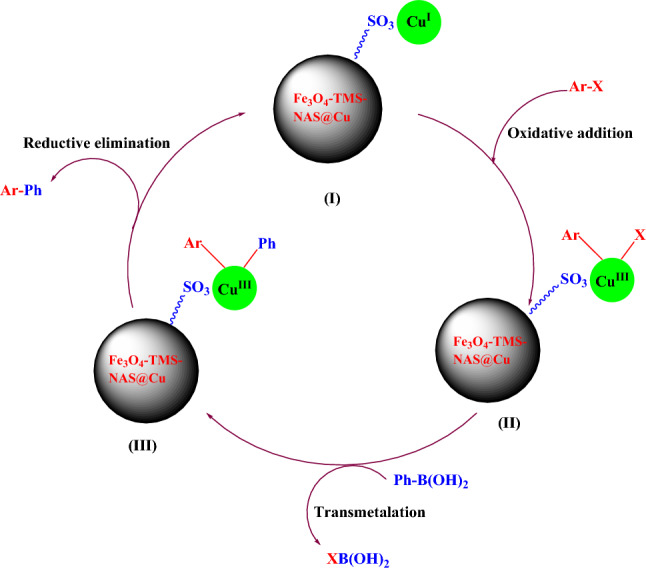
Possible mechanism of the Suzuki–Miyaura coupling reaction.

The suggested mechanism for the Suzuki coupling reaction using Fe_3_O_4_-PTMS-NAS@Cu is demonstrated in Scheme 3. According to the mechanism reported previously^9,24^ the first step is the oxidative addition of copper to the aryl halide that the organocopper intermediate (II) is produced. Next, by transmetallation of (II), intermediate (III) is formed. Reductive elimination of the intermediate (III) led to the formation of product and regeneration the copper catalyst (I) which can be continue the catalytic cycle.

### Reusability of the catalyst

**Figure 10:**
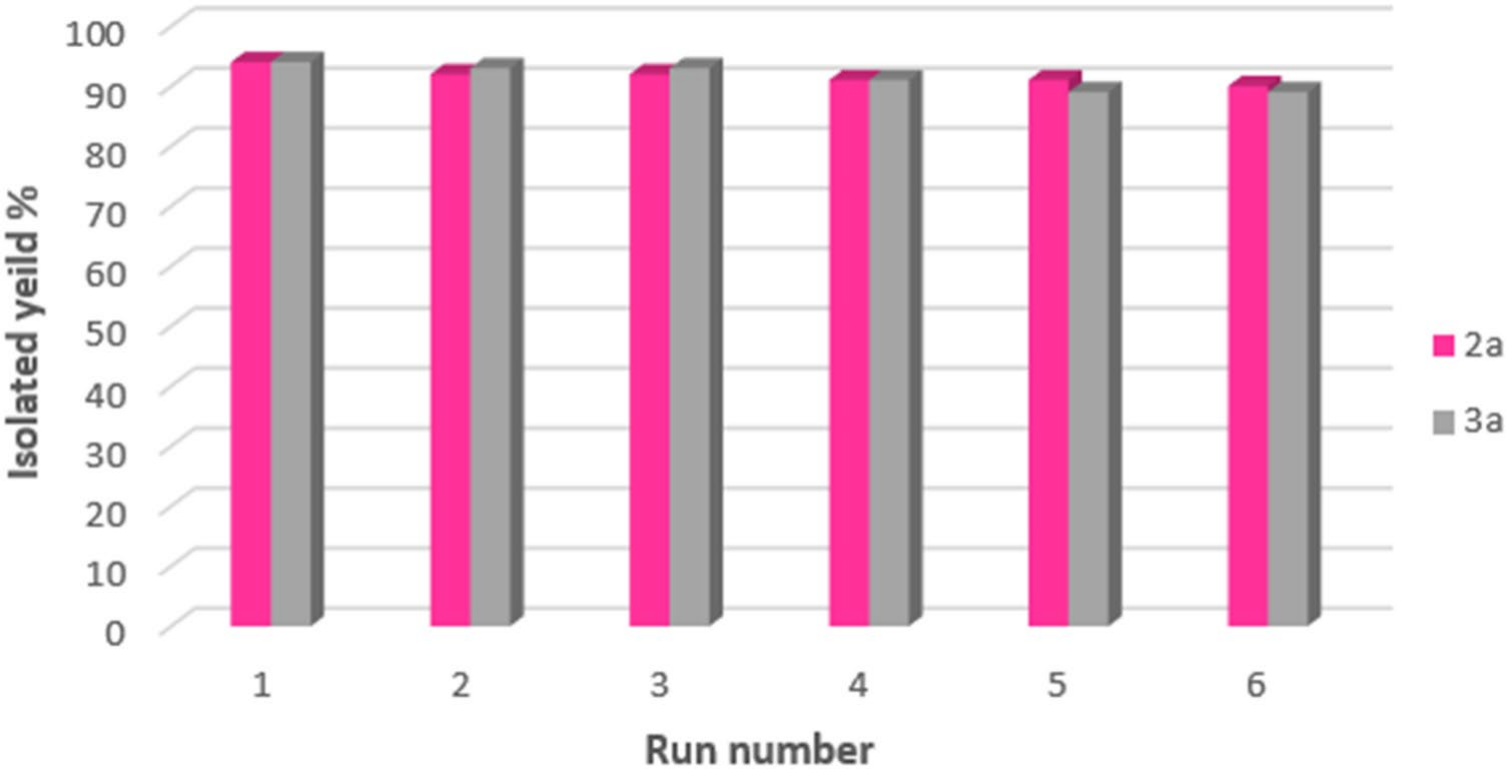
Recyclability of Fe_3_O_4_-PTMS-NAS@Cu MNPs in the synthesis of compounds (**2a**) and (**3a**).

One of the most significant features of magnetic nanoparticles is the capability of being recovered and reused several times. In this regard, after completion of the reaction, the catalyst was separated from the reaction mixture using an external magnet, washed with EtOAc, dried at room temperature and, then, prepared for the next run. We have studied the activity of Fe_3_O_4_-PTMS-NAS@Cu MNPs in the synthesis of compounds (**2a**) and (**3a**) after recovery. The results illustrated that this magnetic nanocatalyst can be recovered and reused for six runs, while maintaining its catalytic activity (Fig. 10).

### Leaching study of the catalyst

In order to consider the heterogeneous nature of Fe_3_O_4_-PTMS-NAS@Cu MNPs in the Stille coupling reaction conditions, hot filtration experiment was performed in the coupling of 4-iodotoluene with triphenyltin chloride. In this study, in half‐time of the reaction (the reaction time is 190 min), 56% of product was obtained. Moreover, this reaction was repeated and in half‐time of the reaction (after 95 min), the catalyst was separated and, then, the filtrated solution was permitted to continue the reaction without the catalyst for a further 95 min. Thereupon, only 58% of 4-Methyl-1,1′-biphenyl as a product was obtained. These experiments confirm that there is no detectable increase in the product concentration, which might be an evidence for a heterogeneous mechanism during the recycling process.

### Characterization of recycled Fe_3_O_4_-PTMS-NASGRONAS@Cu MNPs

**Figure 11:**
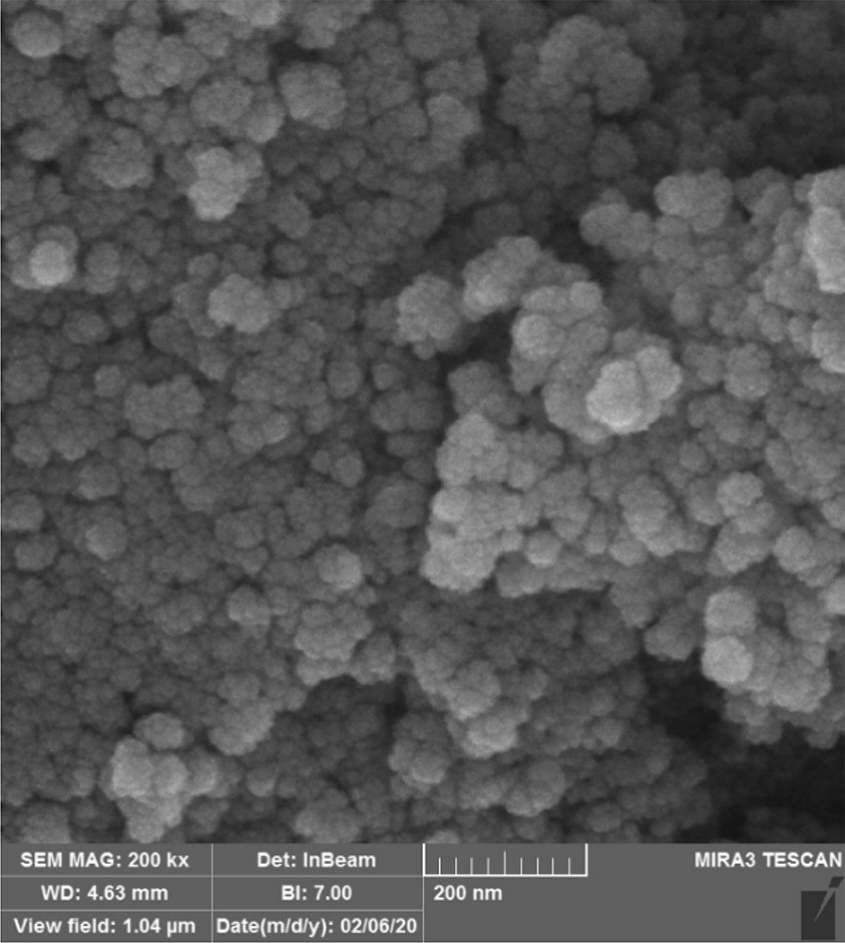
SEM image of Fe_3_O_4_-PTMSNAS@Cu MNPs after recovery.

**Figure 12:**
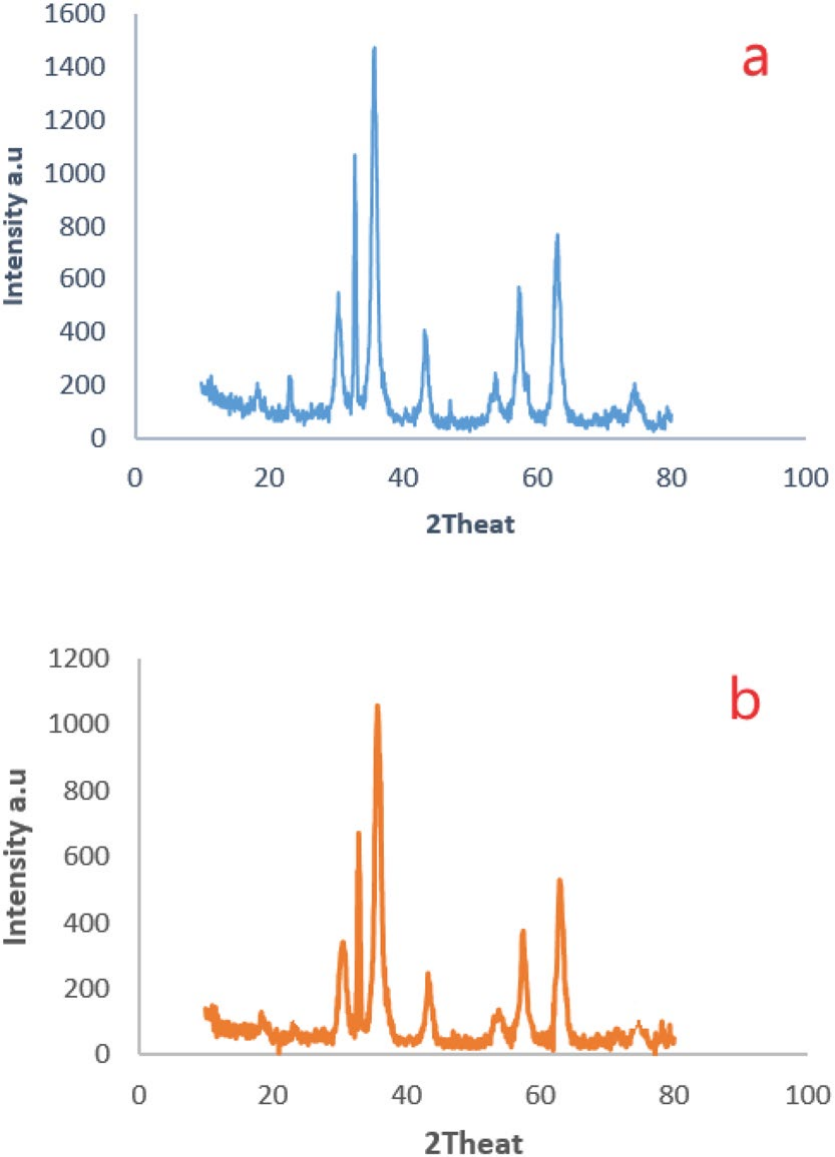
The XRD patterns of Fe_3_O_4_-PTMSNAS@Cu MNPs (**a**) and Fe_3_O_4_-PTMSNAS@Cu MNPs after recovery (**b**).

Fe_3_O_4_-PTMS-NAS@Cu MNPs was recycled up to six runs and, then, characterized using SEM and XRD analysis. The SEM image (Fig. 11) after recovery proved the structure of Fe_3_O_4_-PTMS-NAS@Cu MNPs. In addition, XRD patterns of the fresh Fe_3_O_4_-PTMS-NAS@Cu after recovery are shown in Fig. 12, confirming the structure of the catalyst after recovery.

### Comparison of the catalyst

The efficiency of Fe_3_O_4_-PTMSNAS@Cu was investigated by comparing our results with the previously reported methods in the coupling of iodobenzene with phenylboronic acid and triphenyltin chloride in the synthesis of **2a** and** 3a** products. As shown in Table 4, Fe_3_O_4_-PTMSNAS@Cu has good results, as compared to the other catalysts. Moreover, Fe_3_O_4_-PTMSNAS@Cu possesses several advantages; including, low price, stability, non-toxicity, reaction speed, high yield and easy separation.

**Table 4:** Comparison results of Fe_3_O_4_-PTMSNAS@Cu with other catalysts in the synthesis of **2a** and **3a** products.

Entry	Catalyst	Product	Time (min)	Yield (%)	References
1	Pd/Al_2_O_3_	2a	300	60	^25^
2	Mag-IL-Pd	2a	540	95	^26^
3	Pd-MOT	2a	300	75	^25^
4	NAS@Cu	2a	120	97	^9^
5	Fe_3_O_4_-PTMS-NAS@Cu	2a	90	94	This work
6	SBA-15-EDTA-Pd	3a	300	94	^27^
7	mpg-C_3_N_4_-Pd	3a	720	97	^28^
8	Fe_3_O_4_@SiO_2_@NHC^SPh-Pd(II)	3a	360	96	^29^
9	Fe_3_O_4_-PTMS-NAS@Cu	3a	90	94	This work

## Experimental section

### Apparatus and materials

The chemicals were purchased from Merck and Fluka Chemical Companies, phenylboronic acid (95%), triphenyltin chloride (95%), aryl halides (98%) and natural asphalt was bought from the Kimia Bitumen Zagros Cooperative, Iran. The reactions were monitored with TLC on silica-gel Polygram SILG/UV254 plates. Fourier-transform infrared spectroscopy (FTIR) was performed using FTIR-8300 spectrometer made by Shimadzu. Proton nuclear magnetic resonance (^1^H NMR) spectroscopy was also performed on Bruker AVANCE DPX-400 and DPX-500 spectrometers. Chemical shifts were reported in ppm relative to TMS as the internal standard. The morphology of the catalyst was investigated by scanning electron microscopy (SEM) using Mira 3-XMU. TEM was performed with Philips CM300 to measure the size of the particles. The elemental composition was determined using EDS and Mira 3-XMU. The exact value of Cu in the catalyst was estimated applying Inductively Coupled Plasma (ICP) (VISTA-PRO, Australia). X-ray diffraction (XRD) was investigated using a Holland Philips X, the thermogravimetric analysis (TGA) curve was recorded using a PL-STA 1500 device manufactured by Thermal Sciences, superparamagnetic properties of the catalyst were measured using VSM (MDKFD) and the analysis of the surface chemical composition of Fe_3_O_4_-PTMS-NAS@Cu MNPs was conducted using the X-ray photoelectron spectroscopy (XPS) (Thermo Scientific, ESCALAB 250Xi Mg X-ray resource) see Supplementary Information.

### Synthesis of Fe_3_O_4_-PTMS-NAS@Cu

#### Synthesis of natural asphalt sulfonic acid (NASA)

Initially, natural asphalt (1 g) was mixed with (5 mL) of the concentrated sulfuric acid and, then, the mixture was stirred at 220 °C for 2 h. Next, the reaction mixture was cooled to room temperature and, then, it was slowly poured into 20 mL of distilled ice water. Finally, the product (NAS) was extracted using filtration, washed with distilled water for the several runs and, then, dried at 100 °C in oven^9^.

#### Synthesis of sodium natural asphalt sulfonate (Na-NAS)

In the next step, (1 g) of the previous stage product (NASA) was added to (20 mL) of NaOH solution (10%) and, then, the reaction mixture was stirred for 1 h at room temperature. After evaporation of the solvent, the product (Na-NAS) was dried at 100 °C in oven^9^.

#### Synthesis of Fe_3_O_4_-CPTMS MNPs

Fe_3_O_4_-CPTM was synthesized according to the previous literature^15^.

#### Synthesis of Fe_3_O_4_-PTMS-NAS MNPs

Regarding the synthesis of Fe_3_O_4_-PTMS-NAS MNPs, a mixture of Fe_3_O_4_-CPTMS (1 g) and Na-NAS (1 g) was added to (40 mL) toluene and, then, the reaction mixture was stirred at 100 °C for 24 h. Finally, the product (Fe_3_O_4_-PTMS-NAS) MNPs was extracted using an external magnet, washed several times with EtOH and, then, dried at room temperature.

#### Synthesis of Fe_3_O_4_-PTMS-NAS@Cu MNPs

Fe_3_O_4_-PTMS-NAS MNPs (1 g) and CuCl (0.2 g) were mixed in EtOH (40 mL) and, then, the reaction mixture was refluxed for 24 h. Next, the synthesized Fe_3_O_4_-PTMS-NAS@Cu MNPs were separated using an external magnet, washed with ethanol for the several runs and, then, dried at 80 °C in the oven.

### General procedure for the Suzuki and Stille coupling reactions using Fe_3_O_4_-PTMS-NAS@Cu MNPs

A mixture of aryl halide (1 mmol), triphenyltin chloride (Ph_3_SnCl) (0.4 mmol) or phenylboronic acid (PhB(OH)_2_) (1 mmol), Cs_2_CO_3_ (1 mmol) and Fe_3_O_4_-PTMS-NAS@Cu MNPs (10 mg) in EtOH (2 mL) was stirred at reflux condition. Progress of the reaction was monitored by TLC. After completion of the reaction, the catalyst was separated from the reaction mixture using an external magnetic field and, then, the product was extracted with ethyl acetate (3 × 10 ml). The solvent was evaporated and, finally, pure biphenyl derivatives were obtained in good to excellent yields.

## Conclusion

In conclusion, a new type of magnetically recoverable nanocatalyst (Fe_3_O_4_-PTMS-NAS@Cu MNPs) was synthesized. The efficiency and activity of Fe_3_O_4_-PTMS-NAS@Cu MNPs as an excellent and highly reusable catalyst were investigated in the Suzuki and Stille coupling reactions. In order to characterize this nanocatalyst, various techniques; including, FT-IR, SEM, TEM, EDX, XRD, TGA, VSM, BET, ICP and XPS analysis were used. The XPS analysis confirmed that the natural asphalt has been successfully functionalized. The results obtained from XPS analysis confirmed the presence of sulfonic group, Fe^2+^, Fe^3+^,Si, Cu (I) and Cu (II) in the structure of the catalyst and are in good agreement with the proposed structure of the catalyst. Short reaction times and also good to excellent yields of the products proved the high catalytic activity of Fe_3_O_4_-PTMS-NAS@Cu MNPs. Moreover, this nanocatalyst has low toxicity, can be extracted from the reaction mixture using an external magnet and reused for the several runs while maintaining its catalytic activity (Supplementary Information).

## Supplementary Information

Supplementary Figures.
