# Supplementary material for: XPS and structural studies of Fe3O4-PTMS-NAS@Cu as a novel magnetic natural asphalt base network and recoverable nanocatalyst for the synthesis of biaryl compounds
Source: Sci Rep. 2021 Dec 30;11:24508. doi: 10.1038/s41598-021-04111-z (PMC8718525; doi:10.1038/s41598-021-04111-z)
Supplement: Supplementary file 1 — Supplementary Figures. [file 41598_2021_4111_MOESM1_ESM.docx]

Supporting Information

# [XPS and structural studies of Fe_3_O_4_-PTMS-NAS@Cu as a novel magnetic natural asphalt base network and recoverable nanocatalyst for the synthesis of biaryl compounds](https://www.sciencedirect.com/science/article/pii/S0272884219309903)

Homa Kohzadi^a^, Mohammad Soleiman-Beigi*^,a^

*^a^Department of Chemistry, Faculty of Basic Sciences, Ilam University, P.O. Box 69315-516, Ilam, Iran*

*Corresponding author: Mohammad Soleiman-Beigi*

*Tell /Fax number: +98 (843) 2227022*

*E-mail:* [*SoleimanBeigi@yahoo.com*](mailto:SoleimanBeigi@yahoo.com)*,* [*M.SoleimanBeigi@ilam.ac.ir*](mailto:M.SoleimanBeigi@ilam.ac.ir)

**TABLE OF CONTENTS**

**Contents Page**

**Fig 1.** ^1^H NMR spectrum of [1,1'- Biphenyl]-4-carbonitrile 3

**Fig 2**. ^13^C NMR spectrum of [1,1'- Biphenyl]-4-carbonitrile 4

**Fig 3.** ^1^H NMR spectrum 4-Methoxy-1,1'-biphenyl 5

**Fig 4.** ^13^C NMR spectrum of 4-Methoxy-1,1'-biphenyl 6

**Fig 5.** ^1^H NMR spectrum 4-Nitro-1,1'-biphenyl 7

**Fig 6.** ^13^C NMR spectrum of 4-Nitro-1,1'-biphenyl 8

Fig 7. ^1^H NMR spectrum of 1,1'-Biphenyl 9

Fig 8. ^13^C NMR spectrum of of 1,1'-Biphenyl 10


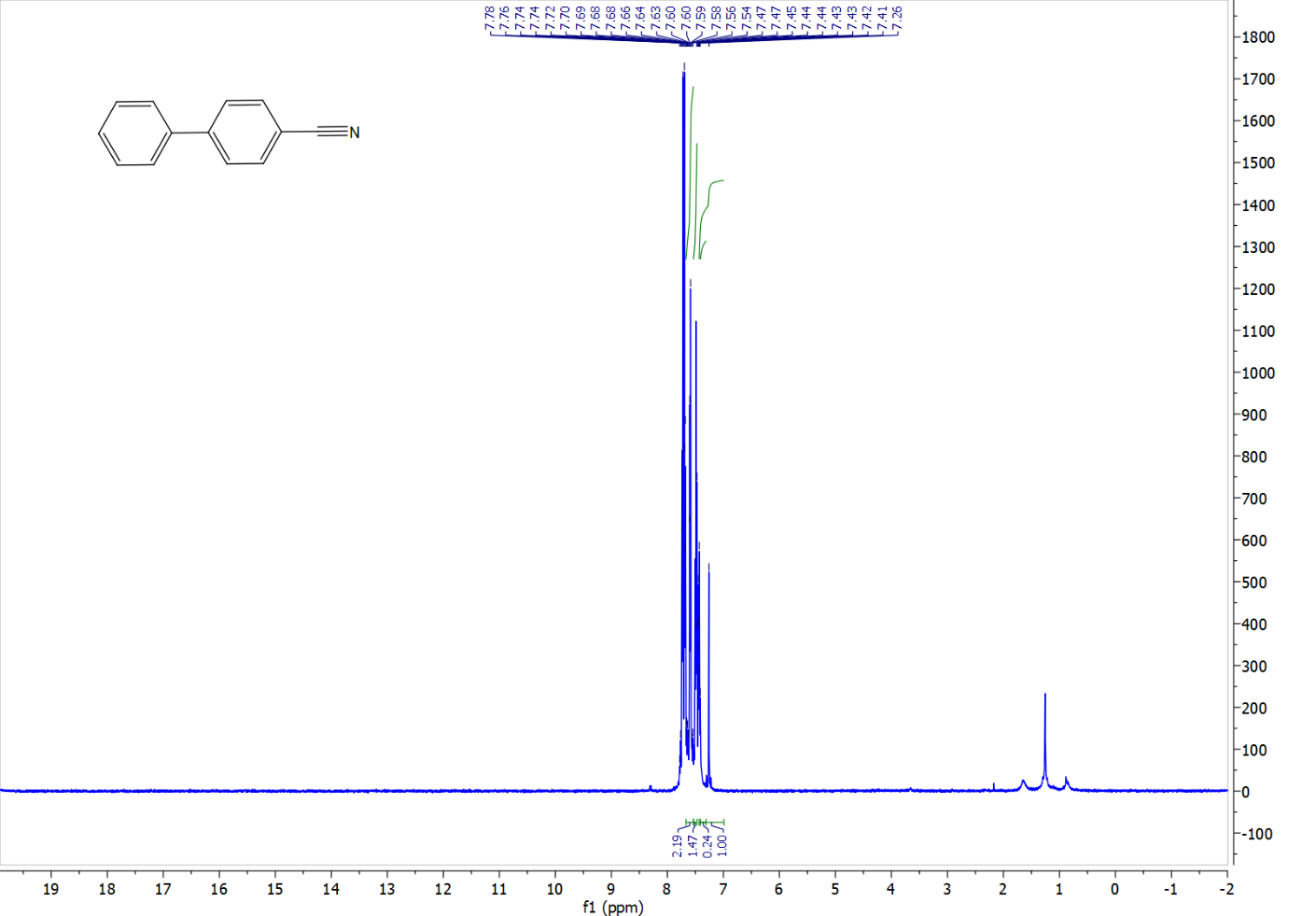


**Fig 1**. ^1^H NMR spectrum of [1,1'- Biphenyl]-4-carbonitrile


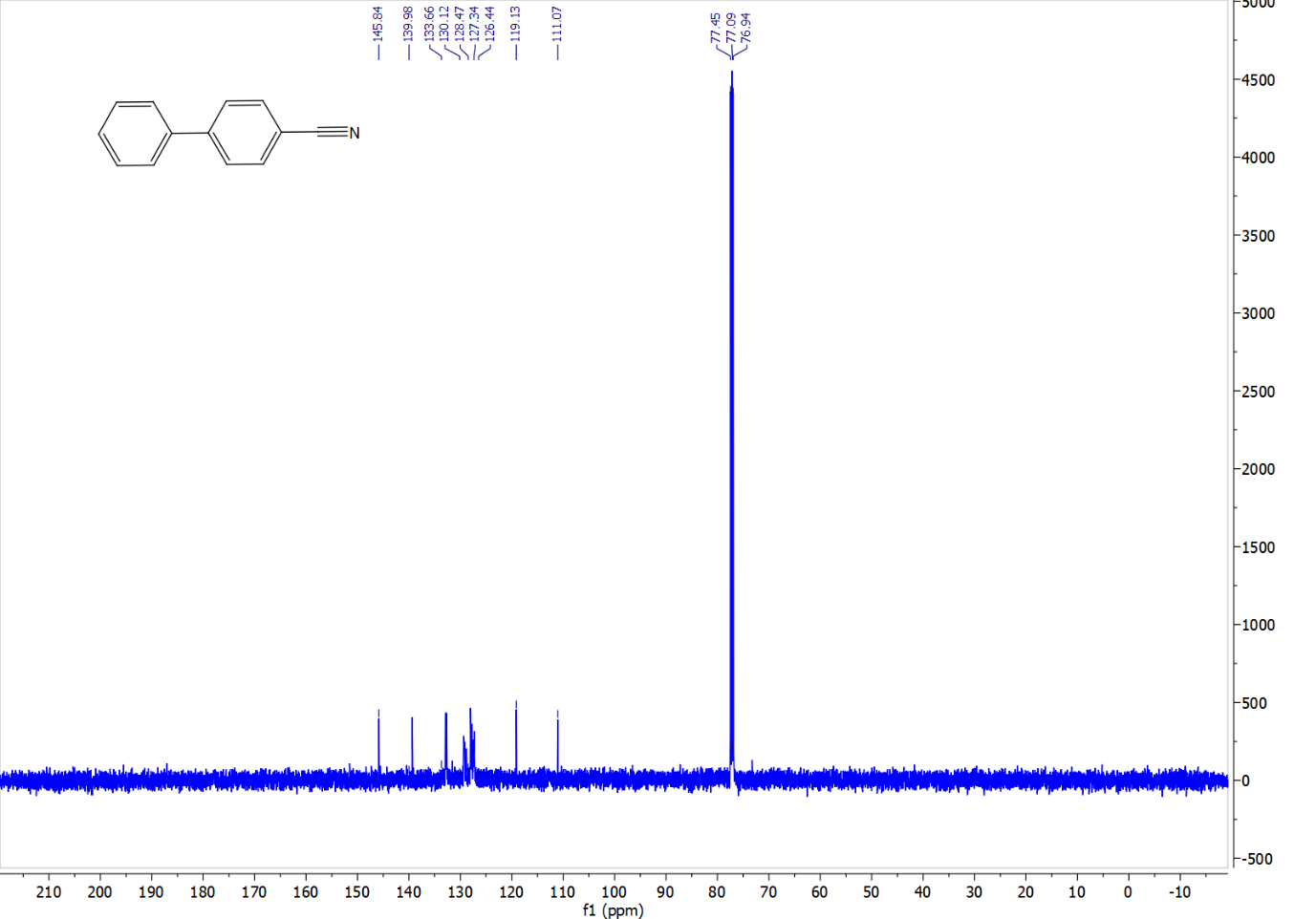


**Fig 2**. ^13^C NMR spectrum of [1,1'- Biphenyl]-4-carbonitrile


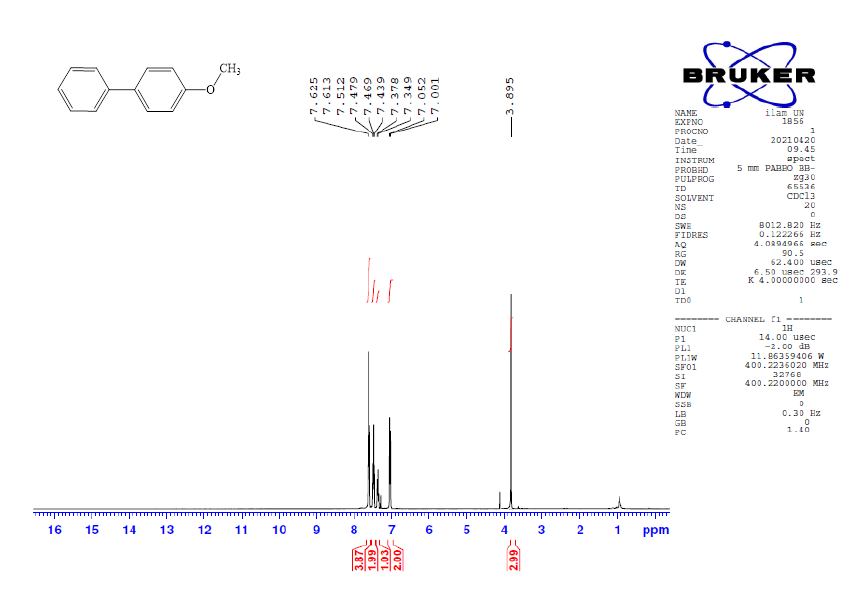


**Fig 3.** ^1^H NMR spectrum 4-Methoxy-1,1'-biphenyl


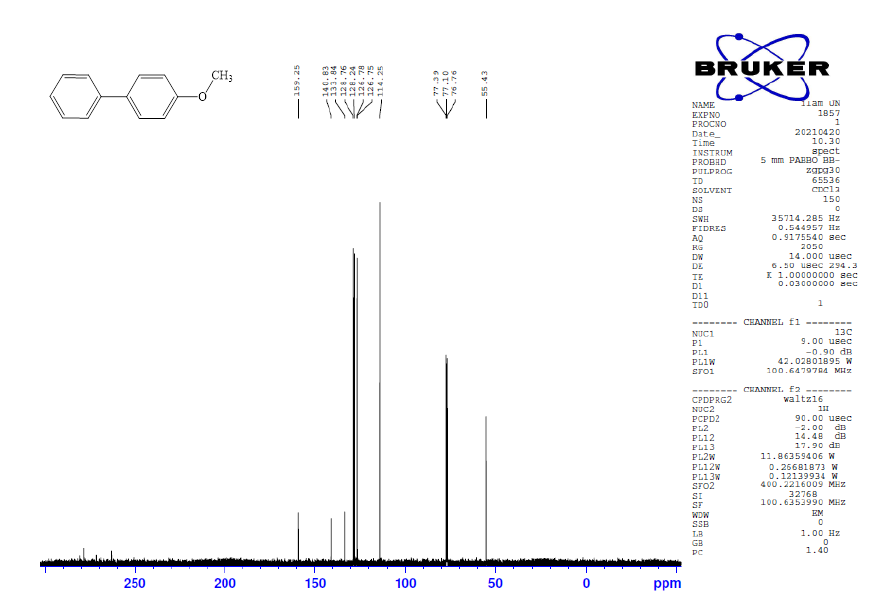


**Fig 4.** ^13^C NMR spectrum of 4-Methoxy-1,1'-biphenyl

**Fig 5.** ^1^H NMR spectrum 4-Nitro-1,1'-biphenyl

**Fig 6.** ^13^C NMR spectrum of 4-Nitro-1,1'-biphenyl


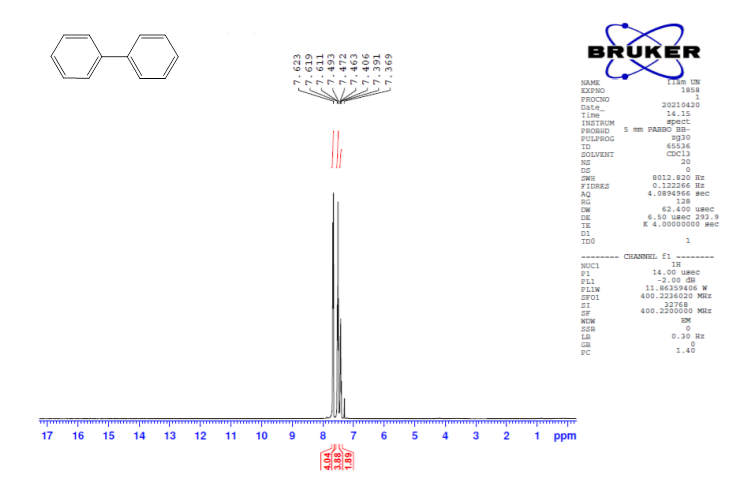


**Fig 7.**^1^H NMR spectrum of 1,1'-Biphenyl


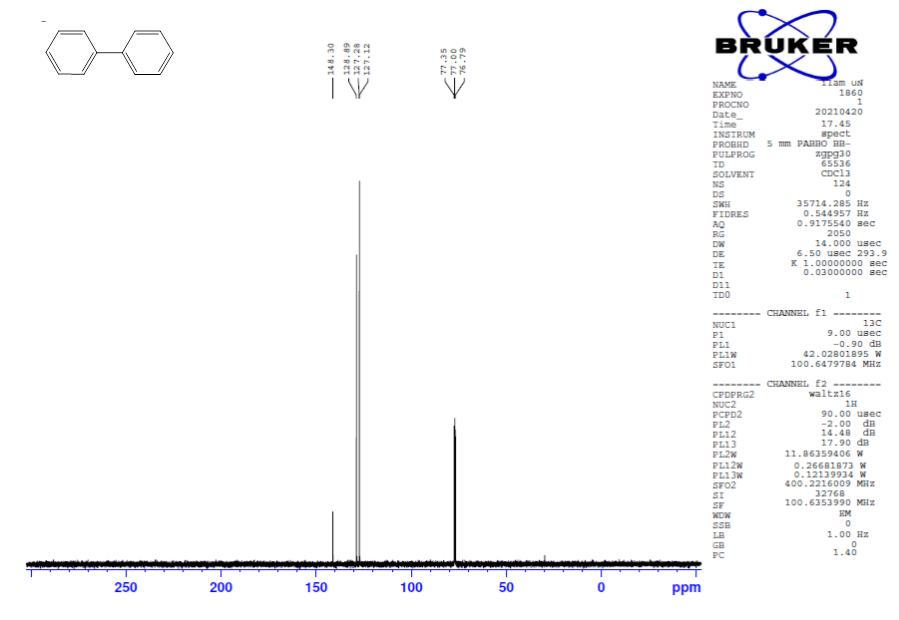


**Fig 8.** ^13^C NMR spectrum of 1,1'-Biphenyl
